# The impact of teleworking on employee idea generation: a self-determination theory perspective

**DOI:** 10.3389/fpsyg.2026.1855286

**Published:** 2026-08-28

**Authors:** Xiaofang Chen, Yinge Chen, Dan Hu, Huili Ye, Mingze Li

**Affiliations:** 1 School of Management, Wuhan University of Technology, Wuhan, China; 2 China Urban Construction Design & Research Institute, Beijing, China; 3 School of Public Administration, China University of Geosciences (Wuhan), Wuhan, China

**Keywords:** empowering leadership, idea generation, remote work, self-determination theory, teleworking, work autonomy

## Abstract

Drawing on self-determination theory, this study examines how teleworking influences employee idea generation and clarifies the motivational mechanism and leadership boundary condition underlying this relationship. We propose that teleworking facilitates employee idea generation by enhancing work autonomy, and that empowering leadership strengthens the extent to which teleworking is experienced as usable autonomy. Study 1 used a between-subjects experimental design and showed that participants in the high remote work condition reported higher creative idea generation than those in the low remote work condition. Study 2 employed a three-wave survey of employees in real organizational settings and found that work autonomy mediated the positive relationship between teleworking and idea generation. Empowering leadership further moderated the teleworking–autonomy relationship and the indirect effect of teleworking on idea generation through work autonomy. These findings extend teleworking and creativity research by identifying work autonomy as a proximal psychological pathway and empowering leadership as a key contextual condition for converting remote-work flexibility into creative benefits.

## 1 Introduction

As digital technologies diffuse, organizational boundaries become more permeable, and work arrangements continue to evolve in the post-pandemic era, teleworking has become an increasingly normalized feature of organizational life (Wang et al., 2021; Nagata et al., 2021). Teleworking changes the pace of task execution, the pattern of interaction, the flow of information, and the visibility through which managers evaluate employees (Hill et al., 2003; Wang et al., 2021). Because these shifts reach the core architecture of work, understanding how teleworking shapes employee behavior has become a central concern in organizational research. Employee idea generation warrants particular attention because it occupies the front end of the innovation process and supplies the raw material for later stages of innovation (Zhou and Shalley, 2008).

Existing research shows that teleworking reconfigures employees' work experiences across multiple domains. Prior studies link teleworking to changes in work design, work engagement, organizational commitment, knowledge sharing, social connectedness, work–family dynamics, and broader employee outcomes (Charalampous et al., 2019; Wang et al., 2021; Nagata et al., 2021; Bermúdez-González et al., 2024). This literature establishes teleworking as a multidimensional change in work context and provides an important basis for examining its implications for innovation-related behavior.

Yet a critical question remains undertheorized: how does teleworking influence employee idea generation? Idea generation requires sustained attention, problem reframing, exploratory search, and the recombination of informational cues into novel possibilities (Amabile, 1988; Zhou and Shalley, 2008). Teleworking can reshape these conditions in competing ways. On the enabling side, distributed work gives employees greater latitude to manage interruptions and sequence tasks, which may support concentration and deeper cognitive processing (Wang et al., 2021). On the constraining side, prior evidence suggests that virtual communication and remote collaboration may weaken the spontaneous exchange and recombination processes that support creativity. Virtual communication can curb creative idea generation (Brucks and Levav, 2022), remote work can make collaboration networks more static and siloed among information workers (Yang et al., 2022), and remote collaboration may fuse fewer breakthrough ideas (Lin et al., 2023). At the same time, lower visibility in remote settings heightens employees' reliance on managerial cues when interpreting whether independent experimentation and deviation from routine practice will be supported (Wang et al., 2021). Teleworking therefore influences idea generation through a broader process of contextual reconfiguration that changes the cognitive, relational, and interpretive conditions under which ideas emerge.

Current research leaves two issues unresolved. First, teleworking has increasingly been treated as a multidimensional work context, yet this insight has rarely been translated into a theory of idea generation. Prior studies document consequences for knowledge sharing, employee experience, and work attitudes, but provide less clarity on which mechanism is most proximal to the psychological conditions that support the generation of ideas (Charalampous et al., 2019; Wang et al., 2021; Bermúdez-González et al., 2024). Second, self-determination theory and creativity research emphasize that self-endorsed action, intrinsic motivation, and perceived control are central to creative behavior (Deci and Ryan, 1987; Zhou and Shalley, 2008; Ryan et al., 2022). In teleworking contexts, however, the process through which structurally available discretion becomes a psychologically usable work experience remains underdeveloped. Employees working remotely interpret whether latitude is legitimate, whether it can be exercised without penalty, and whether it provides a viable basis for directing attention, effort, and exploration (Deci and Ryan, 1987; O'Donoghue and van der Werff, 2022).

**Figure 1:**
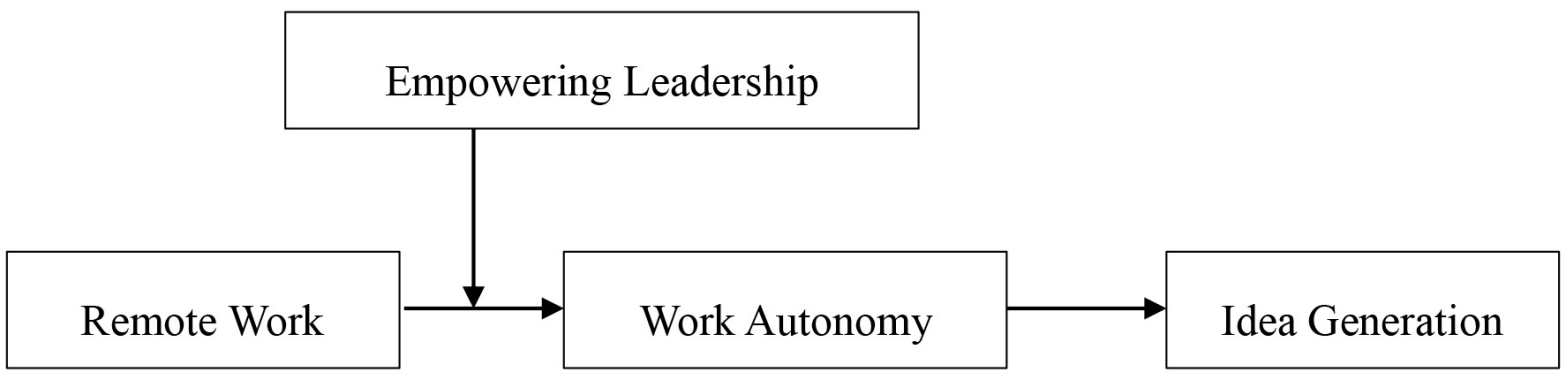
Theoretical model.

Drawing on self-determination theory, we examine the mechanism and boundary condition linking teleworking to employee idea generation (Deci and Ryan, 1987; Ryan et al., 2022). We propose work autonomy as a focal pathway through which teleworking enters the idea generation process. Idea generation requires employees to organize work pace, sustain effort under uncertainty, and engage in exploratory cognitive search, all of which are more likely when employees experience meaningful control over how work is carried out (Deci and Ryan, 1987; Zhou and Shalley, 2008). We further argue that this pathway depends on leadership context. In remote work settings, empowering leadership supplies interpretive cues about whether autonomous action is expected and supported. Through authority sharing, encouragement of participation, expressions of confidence, and outcome-oriented evaluation, empowering leaders help employees treat the discretion released by teleworking as legitimate and usable for self-directed effort and idea exploration (Cheong et al., 2019; O'Donoghue and van der Werff, 2022). We test this framework with an experiment and a multi-wave field study. The proposed theoretical model is presented in Figure 1.

This study contributes in three ways. First, it extends research on teleworking outcomes by bringing employee idea generation more clearly into the teleworking literature, which has more often focused on work design, engagement, commitment, knowledge sharing, and employee experience (Wang et al., 2021; Nagata et al., 2021; Bermúdez-González et al., 2024). Second, it deepens the application of self-determination theory in distributed work settings by clarifying how structurally available discretion is translated into a psychologically usable experience of work autonomy that supports idea generation (Deci and Ryan, 1987; Ryan et al., 2022). Third, it identifies empowering leadership as a boundary condition on the teleworking–autonomy link and shows how managerial signals help convert structural flexibility into a psychological resource for idea generation (Charalampous et al., 2019; Cheong et al., 2019; O'Donoghue and van der Werff, 2022).

## 2 Hypotheses development

### 2.1 Teleworking and idea generation

Teleworking reconfigures the context in which employees perform their work. By loosening temporal and spatial constraints, teleworking gives employees greater discretion over when, where, and how to carry out their tasks (Hill et al., 2003; Wang et al., 2021). This contextual shift is theoretically important for idea generation because generating ideas requires sustained cognitive engagement, problem reframing, and exploratory search beyond routine responses (Zhou and Shalley, 2008). Employees are more likely to engage in these processes when their work environment allows them to regulate interruptions, align task pacing with their cognitive rhythms, and devote uninterrupted attention to complex problems (Wang et al., 2021; Naotunna and Priyankara, 2020).

Self-Determination Theory provides a useful basis for explaining why teleworking is likely to facilitate idea generation. SDT suggests that work contexts supporting volition and self-direction foster higher-quality motivation and more proactive forms of engagement (Deci and Ryan, 1987; Ryan et al., 2022). Teleworking expands employees' latitude in organizing work schedules, selecting work settings, and structuring task execution, thereby increasing the extent to which employees can experience themselves as initiators of their own work behavior (Gajendran and Harrison, 2007; Wang et al., 2021). Under such conditions, employees are more likely to invest effort in experimentation, pursue non-routine solutions, and persist in cognitive search under uncertainty, all of which are central to idea generation (Zhou and Shalley, 2008).

Teleworking may also impose relational and informational constraints on idea generation. Prior research shows that virtual communication can reduce creative idea generation by narrowing visual attention and limiting cognitive focus on the partner (Brucks and Levav, 2022). Evidence from information workers also suggests that remote work can reduce cross-group collaboration and make collaboration networks more siloed (Yang et al., 2022). At a broader scientific-collaboration level, remote collaboration has been associated with fewer breakthrough idea combinations (Lin et al., 2023). These findings indicate that teleworking may weaken the serendipitous interaction and cross-boundary knowledge recombination from which some ideas emerge. However, these findings mainly identify the relational and recombinatory costs of remote or virtual collaboration; they do not necessarily imply that teleworking uniformly suppresses individual idea generation. Idea generation also depends on sustained attention, self-regulated effort, information integration, and active exploration. In knowledge-intensive work, teleworking can strengthen these self-regulatory conditions by reducing workplace interruptions and allowing employees to organize work in ways that better fit task demands and personal cognitive preferences (Wang et al., 2021; Naotunna and Priyankara, 2020). Accordingly, although teleworking may introduce relational frictions, its net effect on employee idea generation is expected to be positive when idea generation relies heavily on self-directed cognitive effort and flexible task organization.

Taken together, teleworking creates a work context that is more conducive to concentrated attention, self-directed effort, and exploratory thinking. These features increase the likelihood that employees will generate novel and useful ideas in the course of their work. Therefore, we propose:

***H1:*** *Teleworking has a significant positive effect on employee idea generation*.

### 2.2 The mediating role of work autonomy

Teleworking may influence idea generation through multiple pathways. The present study focuses on work autonomy because it is the mechanism most proximal to the self-directed regulation of effort, attention, and task execution emphasized by self-determination theory (Deci and Ryan, 1987; Ryan et al., 2022). In organizational settings, work autonomy refers to employees' perceived control over work scheduling, procedures, and methods. It captures whether discretion is experienced as a usable basis for action rather than as a merely formal feature of the work arrangement.

Teleworking expands the objective space for discretion by loosening temporal and spatial constraints. Employees can decide when to perform tasks, how to sequence them, and which environments best support concentration. These changes increase the likelihood that employees experience greater control over how work is conducted, thereby strengthening work autonomy (Gajendran and Harrison, 2007; Deci and Ryan, 1987). This distinction matters theoretically. Structural discretion becomes consequential for behavior when employees interpret it as legitimate and actionable. When teleworking allows employees to regulate pacing, choose procedures, and align work with personal rhythms, work demands are more likely to be experienced as self-endorsed than externally imposed. That shift should heighten perceived work autonomy (Deci and Ryan, 1987; Spector, 1986).

Work autonomy, in turn, should promote idea generation. Creative work depends on intrinsic motivation, flexible problem representation, and sustained exploration of alternative solutions (Amabile, 1988; Zhou and Shalley, 2008). Employees who experience greater control over how work is carried out are more likely to sustain self-endorsed motivation, persist under uncertainty, and invest effort in exploratory problem solving (Deci and Ryan, 1987; Ryan et al., 2022). These conditions are central to idea generation because novel ideas often emerge through prolonged search, experimentation, and the willingness to depart from habitual responses (Amabile, 1988; Zhou and Shalley, 2008). Work autonomy also shapes the allocation of cognitive resources. Employees with higher autonomy devote less attention to complying with externally imposed routines and more attention to framing problems, seeking diverse information, and combining ideas in novel ways. In this sense, work autonomy links teleworking to both the motivational and cognitive foundations of idea generation. We therefore expect teleworking to increase work autonomy, and work autonomy to carry the effect of teleworking to employee idea generation. Accordingly, we propose:

***H2:*** *Teleworking has a significant positive effect on work autonomy*.
***H3:*** *Work autonomy mediates the relationship between teleworking and employee idea generation*.

### 2.3 The moderating role of empowering leadership

Teleworking creates structurally available discretion, yet employees do not automatically translate this discretion into felt autonomy. In remote work settings, the use of flexibility is interpreted through social cues. Employees decide whether autonomous adjustment of schedules, procedures, and problem-solving approaches will be viewed as responsible self-management or as undesirable deviation from expectations.

This interpretive problem makes leadership especially salient. Within self-determination theory, autonomy is supported through social environments that signal trust, acknowledge choice, and reduce controlling pressure (Deci and Ryan, 1987; Ryan et al., 2022). Empowering leadership is closely aligned with this logic because it involves authority sharing, encouragement of participation, confidence in employees' capabilities, and outcome-oriented evaluation (Cheong et al., 2019; O'Donoghue and van der Werff, 2022). In high empowering leadership contexts, employees are more likely to view teleworking discretion as legitimate and safe to use. Supportive leader behaviors communicate that independent judgment is expected, that self-directed action will be trusted, and that flexibility can be used to organize work more effectively. These cues should strengthen the positive effect of teleworking on work autonomy (Cheong et al., 2019). In low empowering or controlling contexts, the same structural discretion may remain psychologically constrained. Reduced visibility can heighten concern about evaluation, prompt employees to self-limit the use of flexibility, and weaken the extent to which teleworking is experienced as genuine autonomy. Under such conditions, the autonomy-enabling potential of teleworking should be less fully realized. We focus on empowering leadership because it operates directly on employees' interpretation of whether available discretion can be exercised. Other boundary conditions, such as task interdependence or organizational context, may also influence remote work effectiveness. Their effects, however, are more distal to the focal psychological process examined here and less directly tied to employees' interpretation of autonomy in everyday work situations.

***H4:*** *Empowering leadership positively moderates the relationship between teleworking and work autonomy, such that the positive relationship is stronger under high empowering leadership than under low empowering leadership*.

If empowering leadership strengthens the effect of teleworking on work autonomy, the indirect effect of teleworking on idea generation via work autonomy should also be stronger when empowering leadership is high. Accordingly, we propose:

***H5:*** *Empowering leadership positively moderates the indirect effect of teleworking on employee idea generation via work autonomy, such that the indirect effect is stronger when empowering leadership is high*.

## 3 Study 1: experimental study

### 3.1 Participants and procedure

Study 1 adopted a between-subjects experimental design to systematically examine the effect of different levels of remote work on employee creative idea generation. Participants were recruited through the Credamo online research platform. After being informed that they would participate in a study concerning work arrangements and job performance, participants entered the experiment upon providing informed consent. The experimental procedure was reviewed and approved by the Ethics Committee of Wuhan University of Technology. Participants were informed that their responses would be used only for academic purposes, would remain confidential, and that they could withdraw from the study at any time without penalty.

To ensure data quality, an attention-check question was embedded in the survey to screen out inattentive respondents. A total of 90 participants were initially recruited. After excluding those who failed the attention check, 86 valid responses remained for subsequent analyses.

Participants were randomly assigned to one of two experimental conditions: a high remote work condition or a low remote work condition. The random assignment procedure ensured balance between the two groups with respect to potential individual differences. Descriptive statistics indicated that both experimental groups contained 43 participants (*N* = 43 per group).

After reading and imagining themselves in the assigned work scenario, participants completed the manipulation check measure and the creative idea generation scale in sequence. Demographic information was collected at the end of the survey. The entire experimental procedure was conducted online.

The average age of the final sample (*N* = 86) was 36.48 years (SD = 11.078). In terms of gender distribution, males and females each accounted for 50% of the sample. Regarding education level, 40.7% had a high school degree or below, 37.2% held an associate or bachelor's degree, and 22.1% had a master's degree or above.

### 3.2 Manipulation and measures

The independent variable in this study was the degree of remote work, operationalized through scenario-based manipulation. Following the approach of Golden (2006), two distinct work scenarios were developed to create a clear contrast in perceived remote work intensity.

Participants in the high remote work condition were presented with a scenario implying 100% remote work, designed to induce a high perception of remote work intensity. In contrast, participants in the low remote work condition were presented with a scenario indicating that only 10% of working time (approximately 4 hours per week) was spent working from home, with the majority of time required to be spent in the office. Following prior scenario-based telework research, we used a high-contrast manipulation to create a clear difference in perceived teleworking intensity and thereby strengthen causal identification (Golden, 2006). Specifically, the high remote work condition approximated full teleworking, whereas the low remote work condition represented a predominantly office-based arrangement. This design is appropriate for isolating the directional effect of teleworking intensity under controlled conditions.

To verify the effectiveness of the manipulation, a manipulation check was conducted immediately after the scenario description. Consistent with prior research, the single-item measure developed by Golden (2006) was used: “What percentage of your total weekly working time is spent outside the office?” Participants responded on a percentage scale ranging from 0% to 100%.

The dependent variable was employee creative idea generation, defined as the cognitive behavioral tendency to generate novel and potentially useful ideas, solutions, or approaches in the workplace. Following prior research (e.g., Škerlavaj et al., 2014), eight items from the creativity scale developed by Zhou and George (2001) were used, specifically those focusing on idea generation. All items were measured on a five-point Likert scale (1 = strongly disagree, 5 = strongly agree). Sample items included “I suggest new ways to improve work processes or products/services” and “I often come up with creative solutions to problems.” The scale demonstrated acceptable internal consistency in this study (Cronbach's α = 0.735).

In line with conventions in organizational behavior research, several demographic variables were included as controls to account for potential confounding effects on creative idea generation. These variables included gender (1 = male, 0 = female), age (measured in years), and education level (ordinal: high school or below, associate/bachelor's degree, master's degree or above). All control variables were self-reported and collected at the end of the experimental procedure. These variables were included because they represent core demographic characteristics that are commonly controlled in organizational behavior research and may be associated with differences in cognitive resources, experience accumulation, and work-related idea generation.

### 3.3 Results

#### 3.3.1 Manipulation check

To ensure the effectiveness of the experimental manipulation, an independent-samples *t*-test was conducted to compare perceived remote work intensity between the two conditions. The results indicated a significant difference between the groups, confirming successful manipulation.

Specifically, participants in the high remote work condition reported a significantly higher perceived percentage of weekly working time spent outside the office (M = 99.07, SD = 3.66) compared with participants in the low remote work condition (M = 10.23, SD = 1.53). The independent-samples t-test revealed that this difference was highly significant, *t*_(56.152)_ = 146.902, *p* < 0.001, with a mean difference of 88.84 and a 95% confidence interval of [87.63, 90.05]. These findings provided strong evidence that the manipulation of remote work intensity (high vs. low) was successful and valid.

#### 3.3.2 Hypothesis testing

**Figure 2:**
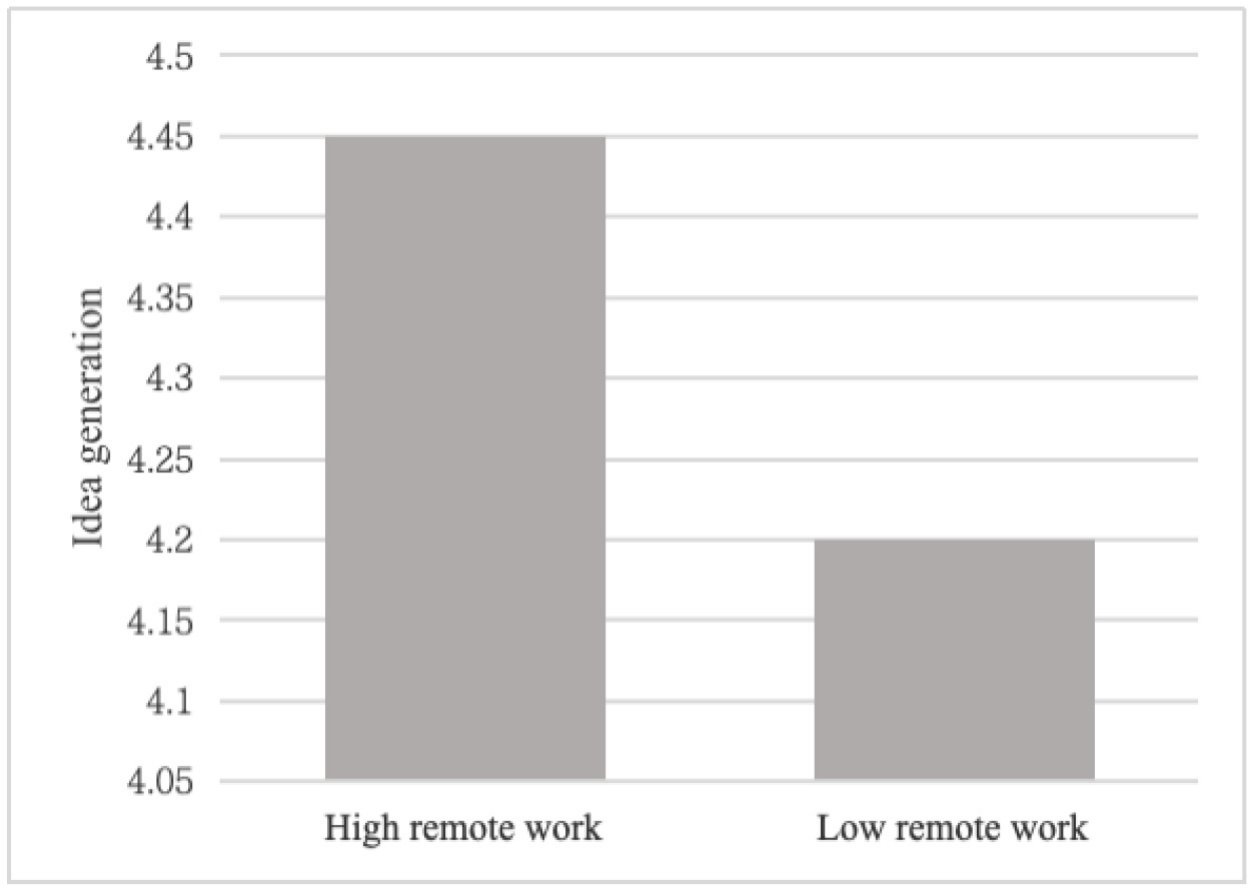
Mean idea generation by teleworking condition.

Hypothesis 1 proposed that remote work would have a significant positive effect on employee creative idea generation. To test this hypothesis, a one-way analysis of variance (ANOVA) was conducted to compare mean levels of creative idea generation between the high and low remote work groups. The difference between the two conditions is illustrated in Figure 2.

Descriptive statistics indicated that participants in the high remote work condition reported higher levels of creative idea generation (M = 4.45, SD = 0.38) than those in the low remote work condition (M = 4.20, SD = 0.18). The ANOVA results showed that the difference between the two groups was statistically significant, *F*_(1, 84)_ = 15.328, *p* < 0.001. The effect-size estimates were partial η^2^ = 0.154 and Cohen's *d* = 0.84, indicating that the manipulation accounted for 15.4% of the variance in idea generation and produced a large, standardized difference between conditions.

These findings indicated that participants assigned to the high remote work condition demonstrated significantly higher creative idea generation compared to those in the low remote work condition. Therefore, Hypothesis 1 was supported.

## 4 Study 2: survey study

### 4.1 Sample and data collection

Although Study 1 provided experimental evidence for a causal effect of teleworking intensity on employee idea generation under controlled conditions, it was limited in capturing the complex processes embedded in real organizational contexts. Moreover, it did not comprehensively test the full theoretical mechanism involving work autonomy and empowering leadership. To enhance ecological validity and examine the proposed moderated mediation model, Study 2 adopted a three-wave field survey in real organizational settings. Temporally separating the focal measures reduced measurement overlap and some concerns about common method variance. However, temporal separation alone does not eliminate omitted-variable bias, reverse causality, or other alternative explanations and therefore does not establish causal identification.

The sample was drawn from three foreign-invested enterprises through the author's professional network. These organizations typically operate under relatively flexible and mature management systems, and their employees commonly engage in remote or hybrid work arrangements. Therefore, they provided a contextually appropriate and heterogeneous sample pool for the present study. This sampling strategy was appropriate for the purposes of the present research because teleworking and hybrid work were already meaningfully practiced in these organizations. As a result, participants were able to report on remote work experiences grounded in their actual work arrangements rather than hypothetical exposure. This feature increased the contextual relevance of the sample for testing the proposed relationships among teleworking, work autonomy, and idea generation. At the same time, the inclusion of three organizations with relatively mature management systems provided a suitable empirical setting for examining the focal model under real organizational conditions.

Prior to data collection, the research team formally communicated with the CEOs of the target organizations to clarify the research objectives, academic value, and confidentiality assurances. After obtaining approval, the HR departments provided employee contact information and assisted in survey distribution. Participation was entirely voluntary and anonymous. The survey procedure was reviewed and approved by the Ethics Committee of Wuhan University of Technology. Prior to participation, employees were informed of the study purpose, confidentiality protections, and their right to withdraw at any time without penalty. Informed consent was obtained before the Time 1 survey.

Data collection followed a strict three-wave design. At Time 1 (T1), participants completed measures of remote work and empowering leadership. Two weeks later, at Time 2 (T2), the same participants completed the measure of work autonomy. Another 2 weeks later, at Time 3 (T3), employee creative idea generation was measured. Participants provided a unique identification code to enable accurate matching across waves while maintaining anonymity. To enhance response rates and ensure completion across all three waves, participants who completed the entire survey process received a monetary incentive of 10 RMB.

At T1, 300 questionnaires were distributed. At T2, 273 valid responses were obtained, yielding a response rate of 91.0%. At T3, 217 employees completed all three waves with successfully matched data, resulting in a final effective sample of 217 participants and an effective response rate of 72.3%. The final sample demonstrated adequate demographic diversity. The average age was 36.49 years (SD = 11.025), with a range from 18 to 59 years. The mean organizational tenure was 8.09 years (SD = 5.78). Females accounted for 61.3% of the sample, and males accounted for 38.7%. In terms of education level, 40.6% had a high school degree or below, 37.3% held a bachelor's or associate degree, and 22.1% had a master's degree or above. Overall, the demographic distribution indicated reasonable representativeness and provided a reliable basis for subsequent multivariate analyses. To assess whether attrition across survey waves introduced systematic bias, we compared the final matched analytical sample (*N* = 217) with participants who completed the earlier survey wave but were not included in the final matched sample (*N* = 56). The results showed no significant differences between the two groups in gender, χ^2^(1) = 0.000, *p* = 0.991, education level, χ^2^(2) = 0.945, *p* = 0.623, age, *t*_(85.75)_ = 0.537, *p* = 0.593. These findings suggest that attrition did not produce substantial demographic differences between the retained and dropped-out participants.

### 4.2 Measures

All constructs were measured using well-established scales that have been widely validated in prior research. To ensure linguistic accuracy and cultural appropriateness in the Chinese context, a rigorous back-translation procedure was conducted. Two bilingual doctoral students independently translated the original English scales into Chinese. An English teacher unfamiliar with the original instruments then translated the Chinese version back into English. Three organizational behavior scholars compared the back-translated version with the original scales and resolved discrepancies through discussion. All multi-item measures were assessed using a five-point Likert scale ranging from 1 (strongly disagree) to 5 (strongly agree), unless otherwise specified.

Remote work, the independent variable, was operationalized as the extent to which employees completed their core work tasks outside the traditional central office using information and communication technologies. Consistent with prior research, particularly the single-item measure developed by Golden (2006), participants reported the percentage of their total weekly working time spent outside the office, with responses ranging from 0% to 100%. This operationalization captures telework intensity as the proportion of total weekly working time performed outside the central office. A single-item indicator is appropriate when the focal construct is concrete, easily understood, and relatively unidimensional (Fisher et al., 2016). Similar frequency- or percentage-based indicators of telework intensity have been employed in prior telework research. Golden (2006) used a percentage-based measure of time spent working away from the office, whereas Nagata et al. (2021) measured home-based telework intensity using frequency-based categories. Therefore, the present study adopts a percentage-based indicator to capture the quantitative intensity of teleworking. At the same time, this measure reflects the quantitative intensity of teleworking rather than its qualitative features, such as voluntariness, communication patterns, or the specific configuration of hybrid work.

Work autonomy, the mediating variable, was measured using three items derived from the classic job characteristics framework proposed by Hackman and Oldham (1975). The scale assesses employees' perceived control over work scheduling, procedures, and methods. Sample items include “This job gives me the opportunity to use personal initiative and judgment in carrying out my work” and “This job allows me to decide on my own how to do my work.” The Cronbach's alpha coefficient in this study was 0.780.

Employee creative idea generation, the dependent variable, was measured using eight items selected from the scale developed by Zhou and George (2001), focusing on the generation of novel and useful ideas. Sample items include “I suggest new ways to improve work processes or products/services” and “I often come up with creative solutions to problems.” The internal consistency reliability in this study was 0.848.

Empowering leadership, the moderating variable, was assessed using the 12-item scale developed by Ahearne et al. (2005). This scale measures employees' perceptions of their supervisors' empowering behaviors across four dimensions: enhancing work meaningfulness, fostering participation in decision-making, expressing confidence in high performance, and providing autonomy from bureaucratic constraints. The reliability coefficient in this study was 0.849.

In line with organizational behavior research conventions, gender, age, education level, and organizational tenure were included as control variables. These controls were intended to account for basic individual differences that may relate to employees' work experience, accumulated human capital, and idea generation.

### 4.3 Analytical strategy

Data were analyzed using Mplus 8.3 in combination with SPSS 26.0. The analytical procedure included data cleaning and preprocessing, confirmatory factor analysis (CFA) to assess discriminant validity among the core constructs, and path analysis to test the proposed moderated mediation model. Maximum likelihood estimation was employed. Indirect and moderated mediation effects were tested using bootstrapping with 5,000 resamples. Statistical significance was determined by examining whether the bias-corrected 95% confidence intervals excluded zero. For significant interaction effects, simple slope analyses were conducted at high (+1 SD) and low (−1 SD) levels of empowering leadership. For the moderation analysis, remote work and empowering leadership were mean centered before creating the interaction term to reduce non-essential multicollinearity and facilitate interpretation. The interaction term was then computed as the product of the mean-centered remote work variable and the mean-centered empowering leadership variable. Unless otherwise noted, the regression coefficients reported in the tables are unstandardized regression coefficients (B), rather than standardized beta coefficients.

### 4.4 Results^1^

#### 4.4.1 Descriptive statistics and correlation analysis

Descriptive statistics and Pearson correlation coefficients for the main variables are presented in Table 1. As shown in Table 1, remote work was positively correlated with work autonomy (*r* = 0.401, *p* < 0.01) and idea generation (*r* = 0.345, *p* < 0.01). Work autonomy was also positively associated with idea generation (*r* = 0.480, *p* < 0.01). In addition, empowering leadership was positively correlated with work autonomy (*r* = 0.411, *p* < 0.01) and idea generation (*r* = 0.394, *p* < 0.01). These correlations provided preliminary, non-causal support for the expected relationships among remote work, work autonomy, empowering leadership, and employee idea generation.

**Table 1:** Descriptive statistics of participants' gender, educational level and experimental conditions.

Variable	Category/ code	Frequency (*N*)	Percentage (%)
Gender	female (0)	43	50.0
	Male (1)	43	50.0
	Total	86	**100.0**
Education level	Senior high school and below	35	40.7
	College/bachelor's degree	32	37.2
	Master's degree and above	19	22.1
	Total	86	100.0
Experimental condition	Low remote work (0)	43	50.0
	High remote work (1)	43	50.0
	Total	86	100.0

To further assess the discriminant validity of the key constructs, confirmatory factor analysis was conducted. The CFA results are reported in Table 2. As shown in Table 2, the proposed three-factor model showed acceptable fit to the data, χ^2^/*df* = 1.946, CFI =.937, TLI = 0.905, RMSEA = 0.066, and SRMR = 0.043. This model also fit the data better than the alternative two-factor and single-factor models. Specifically, the two-factor model combining work autonomy and idea generation showed poorer fit, χ^2^/*df* = 3.894, CFI = 0.791, TLI = 0.710, RMSEA = 0.115, and SRMR = 0.074. The two-factor model combining work autonomy and empowering leadership, as well as the single-factor model, also showed substantially worse fit. These results supported the discriminant validity of the focal constructs.

**Table 2:** Descriptive statistics and correlation matrix of the main variables (*N* = 217).

Variable	Mean	SD	1	2	3	4	5	6	7	8
1. Gender	0.390	0.488	1							
2. Age	36.490	11.025	0.021	1						
3. Education level	1.820	0.772	−0.080	−0.006	1					
4. Work tenure	8.086	5.784	0.042	0.872^**^	−0.015	1				
5. Remote work	0.484	0.197	−0.079	−0.038	−0.065	−0.015	1			
6. Work autonomy	3.865	0.844	−0.033	−0.02	−0.001	0.023	0.401^**^	1		
7.Empowering leadership	3.823	0.461	0.010	0.007	0.077	−0.009	−0.009	0.411^**^	1	
8. Idea generation	3.986	0.497	−0.128	−0.066	0.147^*^	−0.015	0.345^**^	0.480^**^	0.394^**^	1

^*^p < 0.05.

^**^p < 0.01.

*N* = 217.

#### 4.4.2 Model testing and results

The regression results for hypothesis testing are presented in Table 3. As shown in Table 3, after controlling for gender, age, education level, and work tenure, remote work was significantly and positively associated with idea generation (B = 0.465, *p* < 0.001). This finding was consistent with Hypothesis 1.

**Table 3:** Results of confirmatory factor analysis.

Model	χ^2^/*df*	CFI	TLI	RMSEA	SRMR
Three-factor model	1.946	0.937	0.905	0.066	0.043
Two-factor model (combining work autonomy and creativity generation)	3.894	0.791	0.710	0.115	0.074
Two-factor model (combining work autonomy and empowerment leadership)	5.017	0.710	0.598	0.136	0.103
Single-factor model (all questions are loaded on one factor)	6.534	0.585	0.446	0.160	0.110

N = 217; Remote work is a single measurement.

Hypothesis 2 proposed that remote work would be positively associated with work autonomy. As reported in Table 4, remote work was significantly and positively associated with work autonomy (B = 1.713, p < .01 in Model 2; B = 1.802, p < .001 in Model 3). These results indicated that employees reporting higher levels of remote work also reported greater perceived work autonomy. Thus, the findings were consistent with Hypothesis 2.

**Table 4:** Indirect effect results.

Path	Moderator variable	Effect	BootSE	95%confidence interval
Remote work→work autonomy→idea generation				
Moderating effect	High (+1 SD)	0.652	0.189	[0.320, 1.058]
	Low (−1 SD)	0.192	0.161	[−0.046, 0.587]
	Difference index	0.499	0.202	[0.113, 0.919]
Mediating effect	Mediating index	0.402	0.181	[0.125, 0.832]

Hypothesis 3 proposed an indirect association between remote work and idea generation through work autonomy. As shown in Table 4, work autonomy was significantly associated with idea generation (B = 0.234, *p* < 0.001), while remote work remained positively associated with idea generation. The bootstrapping results reported in Table 5 further showed that the estimated indirect association through work autonomy was significant, with an estimate of 0.402 and a 95% confidence interval of [0.125, 0.832], which did not include zero. Therefore, the findings were consistent with Hypothesis 3.

**Table 5:** Regression analysis results of hypothesis testing.

Variable	Work autonomy			Idea generation	
	Model 1	Model 2	Model 3	Model 4	Model 5
Control variables					
Gender	−0.064	−0.005	−0.026	−0.122	−0.091
Age	−0.013	−0.01	−0.009	−0.010	−0.007
Education level	−0.002	0.029	−0.040	0.090	0.099^**^
Work tenure	0.025	0.02	0.014	0.017^*^	0.010
Main effects					
Remote work		1.713^**^	1.802^***^		0.465^***^
Mediator variable					
Work autonomy					0.234^***^
Moderator variable					
Empowering leadership			0.756^***^		
Interaction term					
Remote work × empowering leadership			2.127^***^		
*R* ^2^	0.009	0.166	0.386	0.048	0.297
Δ*R*^2^	/	0.157	0.220	/	0.249

^*^p < 0.05.

^**^p < 0.01.

^***^p < 0.001.

*N* = 217; entries are unstandardized regression coefficients (B). Remote work and empowering leadership were mean centered before creating the interaction term.

**Figure 3:**
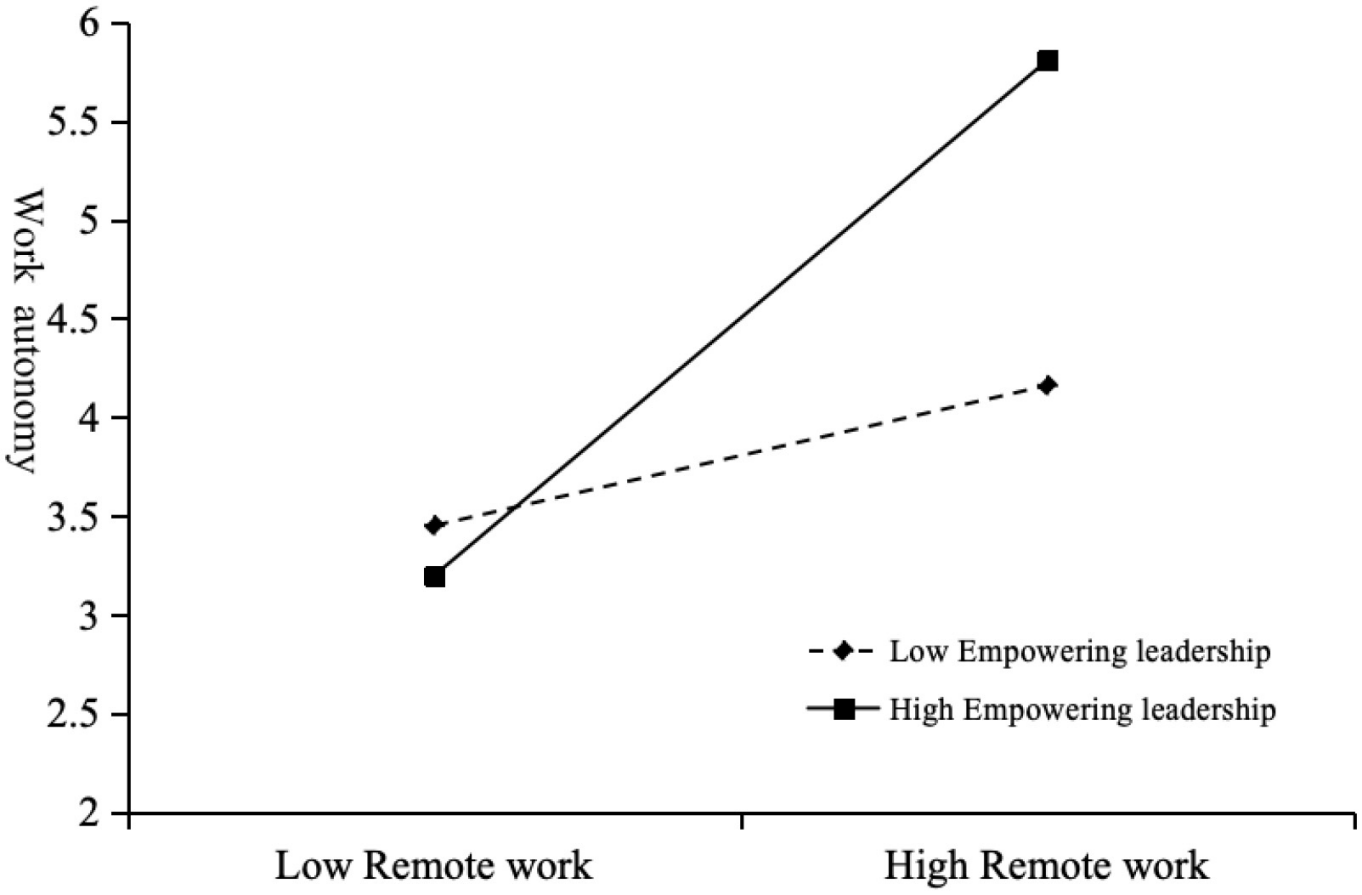
The moderating effect of empowering leadership on the relationship between remote work and work autonomy.

Hypothesis 4 proposed that empowering leadership would moderate the association between remote work and work autonomy. As shown in Table 4, the interaction term between remote work and empowering leadership was significantly associated with work autonomy (B = 2.127, *p* < 0.001). This result indicated that the association between remote work and work autonomy varied according to the level of empowering leadership. The interaction pattern is illustrated in Figure 3. As shown in Figure 3, the positive association between remote work and work autonomy was stronger when empowering leadership was high than when empowering leadership was low. Thus, the findings were consistent with Hypothesis 4.

Finally, Hypothesis 5 proposed a conditional indirect association, such that the estimated indirect association between remote work and idea generation through work autonomy would be stronger under high empowering leadership. The conditional indirect association results are presented in Table 5. As shown in Table 5, the estimated indirect association between remote work and idea generation through work autonomy was significant when empowering leadership was high (Estimate = 0.652, BootSE = 0.189, 95% CI [0.320, 1.058]). However, when empowering leadership was low, the estimated indirect association was weaker and non-significant (Estimate = 0.192, BootSE = 0.161, 95% CI [−0.046, 0.587]). The difference index was also significant (Estimate = 0.499, BootSE = 0.202, 95% CI [0.113, 0.919]), indicating that the indirect association between remote work and idea generation through work autonomy was significantly stronger at higher levels of empowering leadership. Therefore, the findings were consistent with Hypothesis 5.

## 5 Research discussion

### 5.1 Research conclusions

Grounded in the perspective of Self-Determination Theory, this study constructed a moderated mediation model to systematically examine the internal mechanisms and key boundary conditions through which remote work influences employee creative idea generation. Through rigorous empirical designs, including an online experiment and a multi-wave survey study, this research yields several theoretically and practically meaningful conclusions. First, the results confirm that remote work has a significant and positive impact on employees' creative idea generation. Second, the findings clearly demonstrate the critical mediating role of work autonomy in the relationship between remote work and creative idea generation. Finally, the study further reveals the importance of contextual factors, showing that empowering leadership serves as a key boundary condition in optimizing the effectiveness of remote work.

### 5.2 Theoretical contributions

Taken together, the study contributes in three ways. First, the study extends research on the consequences of teleworking by bringing employee idea generation more centrally into the teleworking outcomes literature. Prior teleworking research has mainly examined outcomes such as work design, work engagement, organizational commitment, knowledge sharing, and employee experience (Wang et al., 2021; Nagata et al., 2021; Bermúdez-González et al., 2024). By comparison, whether teleworking supports innovation-related outcomes has remained less settled, with some studies emphasizing constraints on communication and knowledge exchange and others pointing to the benefits of greater flexibility and discretion (Naotunna and Priyankara, 2020). Our findings extend this literature by showing that teleworking can serve as a meaningful antecedent of employee idea generation. In doing so, the study broadens current understanding of teleworking outcomes and places front-end innovation more clearly within the broader conversation about what remote work changes inside organizations.

Second, the study deepens the application of self-determination theory in distributed work settings by specifying how a structural work arrangement becomes a motivationally consequential psychological experience. Existing SDT research has established that self-endorsed action and autonomy support sustain high-quality motivation and creative behavior (Deci and Ryan, 1987; Ryan et al., 2022). Our contribution lies in clarifying that teleworking does not influence idea generation simply because it offers formal flexibility. Its significance lies in creating structurally available discretion that employees can translate into a psychologically usable experience of work autonomy. By making this distinction explicit, the study moves beyond the general claim that autonomy matters for creativity and shows more precisely how a concrete work arrangement enters the motivational process and supports idea generation in distributed work contexts.

Third, the study adds a more relational account of teleworking by identifying empowering leadership as a boundary condition on the teleworking–autonomy link. Broader teleworking research has shown that remote work affects employee outcomes through multiple dimensions, including social connectedness, wellbeing, and organizational attachment (Charalampous et al., 2019; Bermúdez-González et al., 2024). Our findings extend this line of work by showing that, in distributed settings, leadership matters because employees must interpret whether the discretion released by teleworking can be exercised with legitimacy and support. This contribution also extends empowering leadership research (Cheong et al., 2019; O'Donoghue and van der Werff, 2022) by clarifying how managerial signals help convert structural flexibility into a psychological resource for idea generation.

### 5.3 Practical implications

This study offers three practical implications for organizations seeking to use teleworking to support employee idea generation. First, organizations should treat teleworking as a work design choice that needs to fit task characteristics. Teleworking is more likely to support idea generation in roles that require independent thinking, sustained attention, and flexible problem solving. For work with high coordination demands or strong dependence on tacit knowledge exchange, hybrid arrangements may be more appropriate than fully remote arrangements.

Second, organizations should translate remote-work flexibility into usable autonomy through concrete management practices. This means setting clear goals and performance expectations while allowing employees discretion over how they organize their time, pace, and task execution. Managers should reduce excessive process monitoring and create conditions that protect uninterrupted time for deep thinking and idea development.

Third, organizations should view empowering leadership as a necessary condition for effective remote creativity management. Formal teleworking policies alone are unlikely to generate their full benefits unless leaders communicate trust, encourage input, and evaluate employees on outcomes rather than visibility. Leadership development for remote and hybrid settings should therefore focus on autonomy-supportive behaviors that help employees use teleworking discretion productively.

## 6 Limitations and future directions

Several limitations should be noted. Our model explains one pathway through which teleworking shapes idea generation, and this focused design necessarily leaves room for additional mechanisms and boundary conditions.

First, the present study treats work autonomy as a focal mechanism, not an exhaustive explanation. Teleworking may also shape idea generation through relatedness, knowledge sharing, psychological safety, work–family boundary management, cognitive load, and attentional allocation. Future research can examine how these pathways operate alongside work autonomy and whether their relative importance varies across tasks, occupations, and remote-work arrangements.

Second, our treatment of boundary conditions remains selective. We focus on empowering leadership because it is proximal to employees' interpretation of whether teleworking discretion can be exercised. Other influences, including task interdependence, communication norms, digital collaboration infrastructure, and broader organizational climate, may alter the same process from more distal levels. A multilevel perspective would help capture how individual, team, and organizational conditions jointly shape idea generation in remote work settings.

Third, although the present research improves understanding of teleworking and idea generation, some limitations remain in how teleworking was operationalized. In Study 1, teleworking was manipulated as a high-vs.-low dichotomy to maximize contrast and facilitate causal identification, but this design simplifies the fluid nature of contemporary hybrid work arrangements. In Study 2, teleworking was measured using a single-item percentage indicator of time worked outside the office. Such measures can be appropriate when the construct is concrete and unidimensional (Fisher et al., 2016), and similar indicators have been used in prior telework research (Nagata et al., 2021). Even so, this measure captures only the quantitative intensity of teleworking and does not reflect qualitative features such as voluntariness, communication patterns, or hybrid-work configuration. Future research would benefit from more comprehensive measures and more realistic experimental manipulations.

## Data Availability

The raw data supporting the conclusions of this article will be made available by the authors upon reasonable request.

## Footnotes

1 Robustness analysis. We re-estimated the focal models without the demographic control variables. The direction and statistical significance of the key relationships were substantively unchanged. The corresponding model estimates are reported in Supplementary Table S1.
