# Supplementary material for: The impact of teleworking on employee idea generation: a self-determination theory perspective
Source: Front Psychol. 2026 Aug 28;17:1855286. doi: 10.3389/fpsyg.2026.1855286 (PMC13561913; doi:10.3389/fpsyg.2026.1855286)
Supplement: Supplementary file 1 [file Supplementary_file_1.docx]

**Title Page**

**The Impact of Teleworking on Employee Idea Generation: A Self-Determination Theory Perspective**

**Running title:** Teleworking and Employee Idea Generation

Xiaofang Chen^1^, Yinge Chen^1^, Dan Hu^*2^, Huili Ye^3^, Mingze Li^1^

^1^ School of Management, Wuhan University of Technology, Wuhan 430070, China

^2^ China Urban Construction Design & Research Institute Co., Ltd., Beijing 100120, China

^3^ School of Public Administration, China University of Geosciences (Wuhan), Wuhan 430074, China

^*^Corresponding Author

**Name:** Dan Hu

**Affiliation:** China Urban Construction Design & Research Institute

**Full postal address:** No. 36, Deshengmenwai Street, Xicheng District, Beijing 100120, China

**Email:** hudan@cucd.cn

**Telephone:** 13629679701

Declarations

**Conflict of Interest:** The authors declare that they have no competing interests.

**Data Availability:** The data that support the findings of this study are available from the corresponding author upon reasonable request.

**Ethics Approval and Informed Consent:** This study involved human participants and was reviewed and approved by the Ethics Committee of Wuhan University of Technology. All participants provided informed consent prior to participation. Participation was voluntary and anonymous, and participants were informed that they could withdraw at any time without penalty. All procedures were conducted in accordance with the ethical standards of the institutional research committee and with the 1964 Declaration of Helsinki and its later amendments.
